# In vivo and in vitro inhibition of rat liver glutathione transferases activity by extracts from *Combretum zeyheri* (Combretaceae) and *Parinari curatellifolia* (Chrysobalanaceae)

**DOI:** 10.1186/s12906-016-1235-5

**Published:** 2016-07-25

**Authors:** David Gweshelo, Rudo Muswe, Stanley Mukanganyama

**Affiliations:** 1 School of Pharmacy, College of Health Sciences, University of Zimbabwe, Mt. Pleasant, Harare Zimbabwe; 2 Biomolecular Interactions Analyses Group, Department of Biochemistry, University of Zimbabwe, P.O. Box MP 167, Mt. Pleasant, Harare Zimbabwe

**Keywords:** Glutathione transferases, Induction, Inhibition, Natural plant products, Sprague–Dawley rats

## Abstract

**Background:**

*Parinari curatellifolia* and *Combretum zeyheri* are medicinal plants used in Zimbabwe and other Southern African countries for stomach ailments, fever, body aches, wound healing, cancer and tuberculosis. Glutathione transferases (GSTs) are mammalian enzymes that play a significant role in the detoxification and metabolism of many xenobiotic and endogenous compounds and as such can interact with many exogenous compounds including herbal medicines.

The effects of *Parinari curatellifolia and Combretum zeyheri* leaf extracts on glutathione transferases of male Sprague–Dawley rats were investigated in vivo and in vitro after oral administration of either leaf ethanol or water extracts of each plant.

**Methods:**

For *Parinari curatellifolia*, 18 male Sprague-Dawley rats were administered with 0, 500 and 1000 mg/kg body weight of the leaf extracts in corn oil or saline. Animals were sacrificed after 96 h and the kidney and liver samples were removed and used to prepare the cytosolic fractions. GST activity was determined using 1-chloro-2, 4-dinitrobezene. For *Combretum zeyheri*, twenty four male Sprague–Dawley rats were randomly divided into two groups. These two groups were further divided into three groups of four animals each. They were given either the aqueous or ethanol extract at doses of *C. zeyheri* at 0, 50 mg/kg body weight and 200 mg/kg body weight. The extracts were administered orally by oral gavage for four consecutive days and the rats were sacrificed by cervical dislocation on the fifth day.

**Results:**

In animals administered with *C. zeyheri*, GST activity was significantly increased by the 200 mg/kg aqueous extract in the kidneys and livers in vivo whilst the ethanolic extract at 200 mg/kg decreased enzyme activity significantly both organs. Both the ethanol and aqueous extracts inhibited GST activity in vitro with the ethanol extract being more potent inhibitor than ethacrynic acid, a standard GST inhibitor. The increased GST activity in vivo and versus inhibition in vitro suggests that metabolites may be responsible for the effects observed in vivo. For *P. curatellifolia*, a dose-dependent decrease in GST activity was observed in vivo for the animals given the aqueous extract but no changes were observed with the ethanol extract. There was a concentration-dependent inhibition of cytosolic GSTs when *P. curatellifolia* leaf extracts in vitro. The ethanol extract of *P. curatellifolia* exhibited GST-inhibitory activity in the liver with an IC_50_ value of 12 μg/mL and for ethacrynic acid, the IC_50_ was found to be 10 μg/mL. This showed that this extract was a potent inhibitor of GSTs in vitro.

**Conclusions:**

*C. zeyheri* had an inductive effect on GST activity when administered in aqueous solution but inhibited GST in vitro whilst *P. curatellifolia* inhibited GST activity in vivo. Induction of GSTs would be cytoprotective against the toxic effects electrophilic chemicals. Since GSTs are responsible for the synthesis of prostaglandins, the inhibition of GST activity of by these two plants in vivo maybe one of the reasons that makes the plants important for use in the treatment pain and fever in ethnopharmacology.

## Background

Glutathione S-transferases (GSTs) catalyze the nucleophilic attack of the tripeptide glutathione to electrophillic substrates, resulting in addition or substitution reactions depending on the nature of the substrate [1]. Glutathione conjugation is the first step in the mercapturic acid pathway that leads to the elimination of toxic compounds [2].

GSTs have been found to comprise up to 10 % of the total hepatocellular protein, assuring efficient enzymatic conjugation of glutathione to reactive electrophiles [3]. The high concentration of GSTs also provides a reservoir of intracellular binding sites that facilitates noncovalent and sometimes covalent interactions with compounds that are not substrates for glutathione conjugation [4]. The most commonly used co-substrate for GST is a synthetic chemical 1-chloro-2, 4-dinitrobenzene (CDNB) [2] and spectrophotometric assays utilising this substrate are commonly used to report GST activity [5]. Due to their high abundance in the liver, GSTs may be interact with exogenous compounds including natural plant products [6].

GSTs have endogenous functions that include the synthesis of prostaglandins (PG) [7]. PGD_2_ is produced as a major prostaglandin in a variety of tissues and is produced by two distinct prostaglandin D synthases (PGDS) that are hematopoietic (H-PGDS) and lipocallin (L-PGDS). However, production of PGD_2_ in the peripheral tissues and in immune and inflammatory cells is mainly catalysed by H-PGDS, which is a cytosolic, glutathione-dependent enzyme of the sigma class glutathione-S-transferase (GST) family [8]. The GSH-dependent conversion of prostaglandin PGH2 to either PGD_2_ or PGE_2_ are other examples of isomerisation reactions in which GSTs are involved [9]. These reactions do not necessarily involve GST sigma as they involve the mPGES-1, a membrane-associated enzyme with glutathione-dependent activity, and its expression is highly inducible in response to inflammatory stimuli. c-PGES is also a glutathione-dependent enzyme, and is expressed in the cytosol of a wide variety of tissues and cells [10].

Rural households in Zimbabwe rely largely on traditional medicines and with AIDS and a declining economy, there has been an upsurge in the number of people using herbal medicines [11]. *Combretum zeyheri* is a large-fruited bush willow that is found from Congo and Tanzania southwards to South West Africa, Zimbabwe, Botswana, the Transvaal and Natal [12]. The leaves, roots and stem bark of *C. zeyheri* are used medicinally. *C. zeyheri* has been used for reducing symptoms associated with coughs, diarrhea, and cancer [13, 14]. Rheumatism and joint pain is reportedly treated with crushed leaves of *C. zeyheri* [15]. Decoctions of finely ground leaves are reported to be very effective for the treatment of eye inflammation and conjunctivitis. Leaves are pounded with oil and rubbed in for the treatment of back pain or macerated together with roots for the same purpose [16]. The roots and stem bark of *C. zeyheri* are also used for medicinal purposes. Hot water extracts and infusions of the roots and stem bark are added to porridge to treat diarrhea, dysentery and vomiting [17]. Roots are chewed for the treatment of schistosomiasis [17]. Fruits of *Combretum* species are, however, considered poisonous in African traditional medicine [18]. The plant has antibacterial activity against. *Staphylococcus aureus* and antifungal activity against *Candida albicans* [19].

*Parinari curatellifolia* belongs to the family *Chrysobalanaceae* (mobola family/coco plum family) [20]. *Parinari curatellifolia* has various uses in ethnomedicine [21]. *Parinari curatellifolia* had been used in Zimbabwe for constipation, toothaches and for wound healing purposes [11, 22]. The fruit extracts are used to heal inflammation and anemia [23]. *P. curatellifolia* is also used in the treatment of pneumonia, fever, inflammatory conditions and dressings of fractures and dislocations [21]. In Zimbabwe, the leaf extracts and bark have been used as for treatment of skin rashes, tuberculosis, chronic diarrhea, herpes zoster, and herpes simplex [24, 25]. *P. curatellifolia* is also used to treat malaria, wounds, typhoid, fever and has been reported to have antiplasmodic effects [26]. *P. curatellifolia* leaf extracts also demonstrated in vitro antimicrobial activities against two model non-pathogenic species: *Mycobacterium aurum* and *Corynebacterium glutamicum* [25] and against *Corynebacterium ulceran*, *E. coli*, and *Candida albicans* [26].

Approximately 49 % of 877 small molecules that were introduced as new pharmaceuticals between 1981 and 2002 by new chemicals entities were either natural products or semi-synthetic analogs or synthetic products based on natural product models [25, 27, 28]. The most vital contributions herbal medicines have made in developing countries are reducing mortality and morbidity. The main reason for their widespread use is because they are cheap to acquire and most have less toxic effects as compared to the non-herbal medicines and are perceived as safe [29]. Currently efforts are being done to develop new in vitro bench-top bioassays which allow for the practical applications of bioassay-guided fractionations of crude extracts of plants, marine organisms, and microbes in order to discover new pharmaceuticals [30]. One of these aspects is to discover new naturally occurring enzyme inhibitors. There is evidence that administration or consumption of plant-based products can modulate the levels of glutathione transferases in vivo [31]. Epiglottitis, an inflammation of the epiglottis was treated by leaf extracts of *Parinari curatellifolia* [32]. Inflammation is caused by production of prostaglandins and any extract that would have curative effects is likely going to interact with enzymes that produce prostaglandins. Atawodi et al., [33] investigated the effects of *Parinari curatellifolia* root extracts on rats. Their results showed that the plant extracts had significant antioxidant and hepatoprotective effects on acute and chronic liver injuries. *Parinari curatellifolia* flavonoids were also shown to protect rats from acetaminophen–induced hepatic necrosis [23, 34]. The metabolism of acetaminophen produces a reactive quinone metabolite that is detoxified through a glutathione transferase-dependent reaction [4]. It has also been shown that extracts from *C. zeyheri, P. curatellifolia* and *C. platypetalum* have significant antioxidant properties through their abilities to scavenge the nitrite radical in vitro [35]. Chemicals with antioxidant properties have the capacity to induce glutathione transferases in vitro and in vivo through the bZIP transcription factor (Nrf2) and the antioxidant response element (ARE) [36, 37]. Moyo et al., [38] showed that another Combretum species, *Combretum molle* inhibited human recombinant hematopoietic prostaglandin D2 synthase (GST sigma) in vitro, an enzyme that is involved in the synthesis of prostaglandin D2. *Combretum leprosum*-derived triterpenes have been shown to have anti-inflammatory effects in mice [39]. Administration of *Combretum zeyheri* extracts to rats and subsequent isolation of metabolites, showed that the rat liver metabolites could inhibit the growth of microorganisms [40]. Using human recombinant hematopoietic prostaglandin D2 synthase (GST sigma), we have also shown that the *Parinari curatellifolia* leaf extract was the most potent plant extract (IC_50_ = 3.8 μg/ml) to inhibit this enzyme in vitro [41]. The same study also showed that *Combretum zeyheri* methanol and water extract inhibited this enzyme in vitro with IC_50_s of 8 and 29 μg/ml respectively. Based on these studies, the effects of *Combretum zeyheri* or *Parinari curatellifolia* extract in an animal model of glutathione transferase activity was, therefore, warranted. The main objectives of this study was to investigate the effects of *P. curatellifolia* and *C. zeyheri* ethanol and aqueous leaf extracts on glutathione-S-transferases activity in male Sprague–Dawley rats.

## Methods

### Reagents and chemicals

Analytical reagent grade solvents and chemicals of greater than 99 % purity were purchased from Sigma-Aldrich (Steinheim, Germany). The reagents and chemicals used included sodium chloride, trizma base, ethylene diamine tetraacetic acid (EDTA), sodium hydroxide, L-glutathione (reduced), disodium monophosphate, sodium diphosphate, 1 chloro-2, 4 dinitrobenzene (CDNB), ethacrynic acid, sodium carbonate, copper sulphate, potassium sodium tartrate, bovine serum albumin (BSA), Folin and Ciocalteu’s phenol reagent, hydrochloric acid, potassium chloride, corn oil (EEC No 232-281-2), analytical grade ethanol and potassium phosphate dibasic trihydrate.

### Plant collection

Fresh leaves of *P. curatellifolia* were obtained from Centenary (Mashonaland Central Province 16.8000°S, 31.1167°E) whilst those of *C. zeyheri* were obtained from Norton (Mashonaland West Province 17.8833°S, 30.7000°E). The identities of the plants were authenticated by a botanist, Mr. Christopher Chapano, at the National Botanic and Herbarium Garden, Harare, Zimbabwe. Herbarium samples C6E7 and N6E7, were kept at the National Botanic and Herbarium Garden and the Department of Biochemistry, University of Zimbabwe. The leaves were washed with tap water and oven-dried at 50 °C for three days until they were crisp. The leaves were then ground to fine powder using a laboratory blender (Cole Parmer, Vernon Hills, USA).

### Preparation of plant extracts

A volume of 100 mls of analytic reagent grade ethanol was added to 25 g of powdered plant extract. The resultant solution was placed in a rotary incubator (SANYO Gallenkamp PLC, UK) for 6 h. The agitation rate was kept at 150 rev/min. The solution was filtered through a Whatman no 1. filter paper and the procedure was repeated twice. The solution was dried over a fan in open petri dishes for three days. The sample was scraped off with a spatula, weighed and stored at 25 °C for future use. For the aqueous extract, dried leaf powder for each plant of 25 g was boiled in 100 mls of water for 2 h. Water was drained and the residue boiled for a second time under the same conditions. The solution was filtered through a Whatman No. 1 filter paper and the procedure was repeated twice. Finally the extract was dried over a fan. The sample was scraped off with a spatula and transferred to a mortar. The sample was ground with a pestle and mortar until a fine brown powder was formed. The extract was then weighed and stored at 25 °C until when required.

### Experimental animals

The study was approved by the Joint Parirenyatwa Hospital and College of Health Sciences Research Ethics Committee (JREC ref 185/11, Harare, Zimbabwe). Fifty four male Sprague–Dawley rats weighing (90–160 g) were purchased from the Animal House of the University of Zimbabwe. Prior to oral administration with the plant extracts, rats were housed in steel cages five per cage for the aqueous leaf extract treatment and four per cage [42] for the ethanol leaf extract treatment with a bedding of wood shaving that was changed when necessary. The rats were fed with certified food pellets rodent combroids, (National foods (PVT), Ltd., Harare, Zimbabwe) *ad libitum* and allowed free access to tap water. The animal room was maintained in an environmentally controlled facility with an ambient temperature of 25 °C, photoperiod of 12 h light–dark cycle (0600–1800). Animals were maintained and handled according to the recommendations of the good laboratory practice and animal handling (NIH) guidance for the care and use of laboratory animals, Publication No. 85–23, 1985.

### Administration of extracts of *Parinari curatellifolia* and *Combretum zeyheri* to rats

Ethanol leaf extracts were dissolved in corn oil to yield solutions for delivery of 50 and 100 mg/kg body weight (^1^/_140_^th^ and ^1^/_70_th of the LD_50_ respectively). These concentrations were chosen to include the doses used in other studies [43] such that dose-dependent parameters could be inferred. Corn oil was the vehicle and was used as a control so that Sprague–Dawley rats received an equivalent volume to those given *P. curatellifolia* ethanol leaf extracts. A solution of 0.9 % w/v normal saline was used as a control for the water extracts from the plants. Solutions were prepared by dissolving *P. curatellifolia* aqueous leaf extracts in 0.9 % normal saline and these were for delivery of 50 and 100 mg/kg body weight. Expression of xenobiotic metabolizing enzymes such as glutathione transferases can be hormonally regulated [3] and so to rule out the interference from hormonal effects, only male animals were used. Male rats were separated into three groups: 100 mg/kg body weight, 50 mg/kg body weight and corn oil / normal saline. Each group consisted of 5 animals administered with the aqueous extract of *P. curatellifolia* and 4 animals administered with the ethanol extract. The body weights were recorded daily to allow for the appropriate dose of *P. curatellifolia* leaf extract solutions to be administered. All rats were administered with the plant extracts through gastric gavage (6.7 ml/kg) at 24- h intervals to allow for maximal excretion of the previous dose prior to injection. Three days following oral administration of the extracts and control solutions, the rats were starved overnight the body weight body weight determined. The rats were sacrificed by cervical dislocation.

For *C. zeyheri*, male Sprague–Dawley rats were first randomly divided into two groups of twelve, one group as given the ethanol extract whilst the other was given the aqueous extract. Each of the twelve rats were then further divided into three groups, each group containing four animals. The first group was given the drug vehicle alone, the second group was given the 50 mg/kg body weight (B/W) dose and the last group was given the 200 mg/kg BW dose. Administration of these solutions was done by oral gavage for four consecutive days. The weight of each animal was determined before being given the appropriate dose.

### Preparation of cytosolic and microsomal fractions from rat tissue

Throughout the procedure tissue fractions were kept cold (0–4 °C) by using pre-cooled homogenization medium (50 mM Tris–HCl containing 0.154 M KCl) and keeping centrifuge rotors, tissue homogenizer, measuring cylinders, beakers and centrifuge tubes in ice. Excess blood was removed from the liver by perfusing the livers with ice cold normal saline using a Pharmacia LKB pump P-1 (Pharmacia, Uppsala, Sweden) at a flow rate of 50 ml/h through the still beating heart then gently blotting with dry tissue paper. Perfusion was performed *in situ* to remove blood. The livers and kidneys were removed and blotted dry. The liver or kidney samples were, weighed, wet mass recorded and livers quickly transferred to a homogenizing vessel, which was kept, on ice. Homogenizing medium 50 mM Tris–HCl pH 7.4 containing 154 mM KCl was added to each sample (5 ml/g of liver). The liver or kidney samples were cut into suitable pieces and homogenized with a motor driven Potter-Elvehjem homogenizer type R2R (Heidolph Elektro KG., Kelheim, Germany). The homogenates were decanted into measuring cylinders and the final homogenates adjusted to approximately 0.25 g fresh tissue/ml (25 % homogenate). The final volume was recorded and “parafilm ^TM^” (Sigma-Aldrich Co., Steinheim, Germany) was added to cover. Mixing was carried gently and a desired amount transferred to pre-cooled centrifuge tubes. The tubes were capped and a post mitochondria supernatant fraction prepared by centrifugation at 10000 x g (7500 rev/min) for 20 min at 2–4 °C in a Beckman Optima LE-80 k ultracentrifuge (Beckman Instruments Inc., California, USA). The tubes were removed and the supernatant decanted in pre-cooled measuring cylinders. Centrifuging was then done at 105000 x g (35000 rev/min) in a Beckman Optima LE-80 k ultracentrifuge (Beckman instruments Inc., California, USA) for an hour to get the microsomal pellet and supernatant fraction (cytosol GSTs). The supernatant was dispensed into 1.5 ml labeled microtubes and stored at −35 °C. Protein content in the liver and kidney cytosolic fractions was determined using the Lowry method [44].

### Determination of GST activity

The total GST activity in cell supernatants was measured spectrophotometrically using final concentrations of 1 mM 1-chloro-2,4-dinitrobenzene (CDNB) and 1 mM glutathione, as substrates in 0.1 M potassium phosphate buffer pH 6.5, and GST (0.125 mg/ml protein) [2]. Cytosolic glutathione transferases activities were assessed using 0.5 mg/ml and 2 mg/ml enzyme dilutions for the liver and kidney samples respectively. All GST mediated conjugation reactions were carried out at a temperature of 30 °C using a microplate incubator (Jitterbug, 130000, Boekel Industries, Philadelphia, USA). GST mediated conjugation of 1-chloro-2, 4- dinitrobenzene (CDNB) to glutathione (GSH) was measured using 96-well microtitre plates in a microplate reader (Biokinetics reader EL 340 microplate, Highland Park, 998, Winsooki, VT, USA). The reaction was initiated by the addition of CDNB solution to each well.

### In vivo enzyme assay

For the in vivo study, the cytosolic fractions from treated animals were used without the addition of the plant extracts to the reaction mixture. The in vivo enzyme assay incubation mixtures (300 μL) contained 135 or 185 mls 0.1 M potassium phosphate buffer pH 6.5, 15 μL 20 mM CDNB, 100 μL 3 mM GSH, and 50 μL GST enzymes. Potassium phosphate buffer was added to each well followed by cytosolic solution and glutathione. The plate was allowed mixed for a minimum of 5 min at 30 °C. The reaction was initiated by the addition of CDNB solution to each well. After addition of CDNB the microplates was transferred to the reader. The plate was read at 5-min intervals for a total of 15 min in a microplate reader (Biokinetics reader EL 340 microplate, Highland Park, 998, Winsooki, VT, USA). Graphs of time against absorbance were generated for each reaction and from these the rates of enzyme activity were determined.

### In vitro enzyme assay

For the in vitro enzyme assay, incubation mixtures (300 μL) contained 135 or 85 mls 0.1 M potassium phosphate buffer pH 6.5, 15 μL 20 mM CDNB, 100 μL 3 mM GSH, 50 μL plant extracts/ 15 μL ethacrynic acid and 50 μL 2 mg/ml or 0.5 mg/ml GST enzymes. Controls were run in triplicates (for the aqueous extract control group) and quadruplicates (for the ethanol extract control group). Assay buffer was added to each well followed by the cytosolic fraction, plant extract (or ETA) solutions and glutathione. The plate components were mixed in shaker for a minimum of 5 min at 30 °C. The reaction was initiated by the addition of CDNB solution to each well. After addition of CDNB, the plate was transferred to the microplate reader. The plate was read at 5-min intervals for a total of 15 min.

## Statistical analysis

Statistically significant differences among the mean values were determined by one way analysis of variance (ANOVA). A Dunnet’s posttest was used as post test to determine which mean values were significantly different from the control values. All computations were performed using Graph Pad Prism4® software (Version 4.0, Graph pad Software Inc, San Diego, USA).

## Results

### Effects of the plant extracts on physiological parameters

**Fig. 1:**
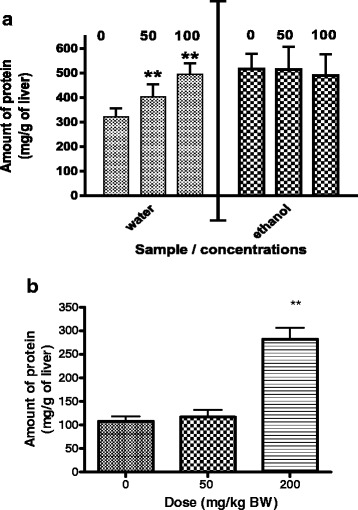
The effects of *P. curatellifolia* and *C. zeyheri leaf* extracts on protein content from rat livers. **a**. The effects of the ethanolic and aqueous *P. curatellifolia leaf extract* on liver protein content. Data are expressed as the mean ± standard error (*n* = 5 for water extract and *n* = 4 for ethanol extract) **b**. The effects of the ethanol leaf extract of *C. zeyheri* on liver protein content. Data are expressed as the mean ± standard error (*n* = 4). *P < 0.01

Administration of the methanol or aqueous extracts of *P. curatellifolia* did not have an effect on the weights of the animals, on organ to body weight ratios (data not shown) but had an effect on the protein content from the liver (Fig. 1a). The water extract increased the protein content in the liver by 26 and 54 % respectively for both the 50 and 100 mg/kg body weight groups. Administration of the ethanol or aqueous extracts of *C. zeyheri* did not have an effect on the weights of the animals, on organ to body weight ratios (data not shown) but produced a significant change of 162 % in total liver protein quantity was observed in the animals treated with 200 mg/kg BW of the *C. zeyheri* ethanol extract (Fig. 1b).

### The effects of *P. curatellifolia* and *C. zeyheri* leaf extracts on GSTs activities in vivo

**Fig. 2:**
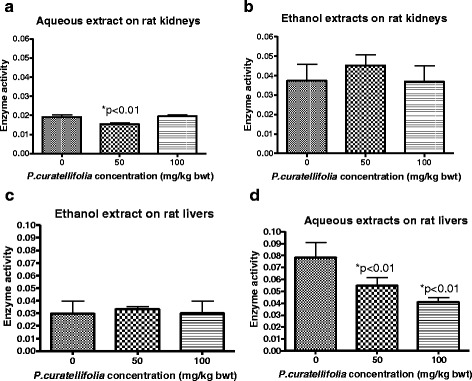
**a.** The effects of *P. curatellifolia* ethanol and aqueous leaf extracts on kidney GSTs activity in vivo. The enzyme activity was determined using CDNB as substrate. **b**. The effects of *P. curatellifolia* ethanol and aqueous leaf extracts on liver GSTs activity in vivo after 72 h. Data are expressed as the mean ± standard error (*n* = 5 for aqueous and *n* = 4 for ethanol extract treated animals). * *P* < 0.01

The effects of *P. curatellifolia* ethanol and aqueous leaf extracts on kidney and liver GSTs activities in vivo are shown in Fig. 2a and b respectively. In the kidneys, the animals that were administered with the ethanol extract, showed no significant differences in enzyme activity between the control and the treated animals. For the animals that were administered with the aqueous extract, there was a decrease by 20 % in enzyme activity for the 50 mg/kg BW group. In the livers (Fig. 2c and d), the ethanol extract treated animals, showed no significant differences in enzyme activity between the control and the treated animals. Different activities were noted on GST activity in the liver and kidneys on animals administered with aqueous extracts. GST activity was reduced by 20 % in the kidneys at 50 mg/kg body weight and 28 % and 42 % for the 50 mg/kg body weight and 100 mg/kg body weight in the liver respectively. Considering rat liver GST activity in the control groups, it was shown that the enzyme activity for the aqueous extract-treated group was 167 % higher than that of the ethanol treated group. Control group comparison shows that the normal saline treatment resulted in higher enzymatic activity as compared to the corn oil treatment.

**Fig. 3:**
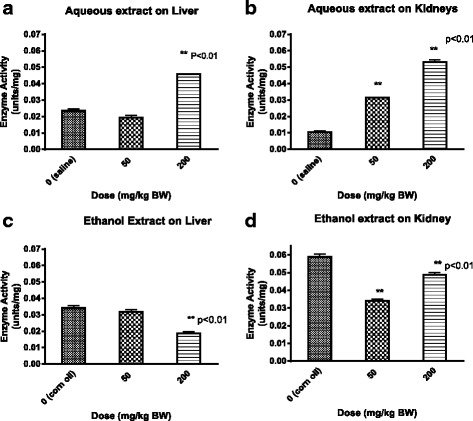
The effect of different *C. zeyheri* doses on the activity of GST compared to the respective control groups. **a** and **b** show the effects of the aqueous and ethanol extracts on liver GSTs respectively whilst **c** and **d** show the effects of the aqueous and ethanol extracts on kidney GSTs respectively. A significant change in GST specific activity is indicated by ** (*p* < 0.01)

For *C. zeyheri* (Fig. 3), an increase in GST activity was observed in the liver and kidney samples from the animals given 200 mg/kg BW aqueous extract (Fig. 3a and b). The GST activity increased by 92 % and 400 % for the liver and kidney samples respectively. The ethanol extract of *C. zeyheri* however, had a different effect on the GSTs in vivo. The 200 mg/kg ethanol extract of *C. zeyheri* caused a decrease in the rat liver and kidney GST activities (Fig. 3c and d) respectively. The GST activity decreased by 45 % and 17 % in the liver and kidney samples respectively. It was also observed that amongst the control animals given only normal saline, there was higher GST specific activity in the livers than in the kidneys, whilst the reverse was observed for the control animals given corn oil alone.

### The effects of *P. curatellifolia* leaf extracts on GSTs activities in vitro

**Fig. 4:**
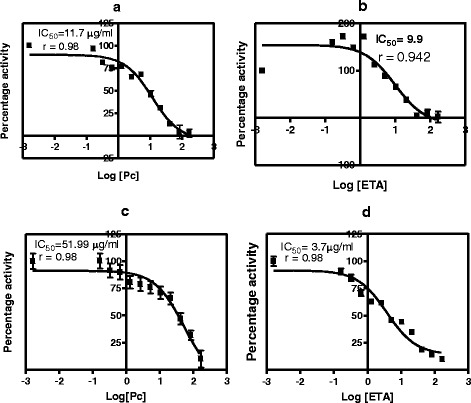
The IC_50_ value determination for ethacrynic acid and *P. curatellifolia* ethanol extracts in liver and kidney samples from corn oil-treated group. **a** Percentage inhibition of liver GSTs activity versus log concentration of *P. curatellifolia* ethanolic extracts. **b** Percentage inhibition of liver GSTs activity versus log concentration of ethacrynic acid (ETA). **c** Percentage inhibition of kidney GSTs activity versus log concentration of *P. curatellifolia* ethanolic extracts. **d** Percentage inhibition of kidney GSTs activity versus log concentration of ETA

**Fig. 5:**
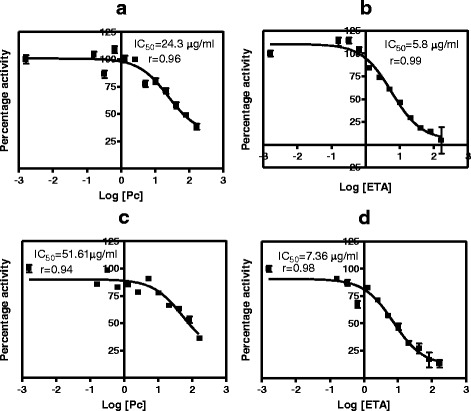
The IC_50_ value determination for ethacrynic acid and *P. curatellifolia* aqueous extracts in liver and kidney samples from normal saline treated group. **a** Percentage inhibition of liver GSTs activity versus log concentration of *P. curatellifolia* aqueous extracts. **b** Percentage inhibition of liver GSTs activity versus log concentration of ETA. **c** Percentage inhibition of kidney GSTs activity versus log concentration of *P. curatellifolia* aqueous extracts. **d** Percentage inhibition of kidney GSTs activity versus log concentration of ETA

The IC_50_ values for *P. curatellifolia* and ethacrynic acid a standard GST inhibitor, were determined by plotting the percentage inhibition of GSTs activity versus log concentration of *P. curatellifolia* and ethacrynic acid. The addition of *P. curatellifolia* leaf extracts to the reaction medium led to a concentration-dependent inhibition of GST activity from in vitro (Fig. 4). In the livers, the percentage inhibition of GSTs activity varied from 38–100 % for the aqueous extract and 3–96 % for the ethanol extract. The inhibition of GSTs activities in the kidneys varied from 36–85 % for the aqueous extract and 10–100 % for the ethanol extract (Fig. 5). For all the plant extracts, the IC_50_ values were determined from such graphs and are shown in Table 1. All the IC_50_ values for the plant extracts were higher compared to those of ETA. The IC_50_ values for the rat livers treated with corn oil had lower values as compared to the normal saline treated group. However, there were no significant differences in the IC_50_ values observed in the GSTS from kidneys that were exposed to the ethanol and aqueous extracts. GST activity from the two organs showed that the IC_50_ value values for GSTs from the kidneys were higher than the IC_50_ values of GSTs from the kidney.

**Table 1:** IC_50_ values for *P. curatellifolia*, *C. zeyheri* and ethacrynic acid determined from direct interactions with GST *in vitro*. The IC_50_ values were determined by reading the concentration at 50 % inhibition from the dose response graphs

Organ	Extract	IC_50_ (μg/ml)	ETA IC_50_ (μg/ml)
	*Parinari curatellifolia*		
Liver	Aqueous	24	6
Liver	Ethanol	12	10
Kidney	Aqueous	52	7.4
Kidney	Ethanol	52	3.7
	*Combretum zeyheri*		
Liver	Aqueous	68	5.4
Liver	Ethanol	1.3	10
Kidney	Aqueous	80	10
Kidney	Ethanol	1.4	3.1

**Fig. 6:**
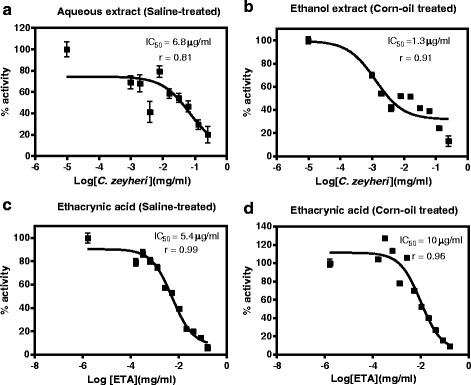
The effect of increasing the concentration of *C. zeyheri* extract or the ethacrynic acid on control liver cytosolic samples from the rats treated with saline or corn oil. The graphs are plots of percentage GST activity against the logarithm of the concentration of the plant extract or ethacrynic acid. Graph **a** and **b** show the effects of different concentrations of the plant extracts (saline–treated or corn oil-treated) on rat hepatic GST percentage activity respectively. Graphs **c** and **d** show the effects of different ethacrynic acid concentrations on the percentage activity liver GSTs from the saline and corn oil control groups respectively

**Fig. 7:**
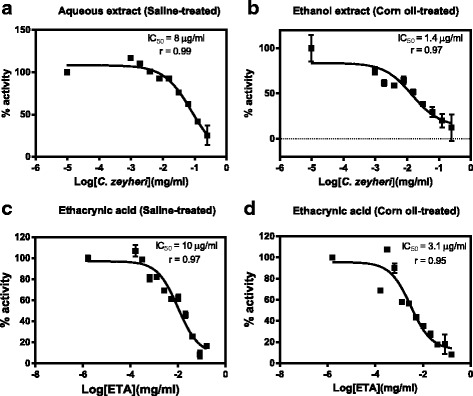
The effect of increasing the concentration of *C. zeyheri* extract or the ethacrynic acid on control kidney cytosolic samples from the rats treated with saline or corn oil. Plots of percentage GST activity versus log concentration of *C. zeyheri* extracts as well as log concentration of ethacrynic acid for the effects of *C. zeyheri* on GST activity in vitro. Graphs **a** and **b** show the effects of the aqueous and ethanol extracts on kidney GSTs respectively whilst C and D show the effects of ethacrynic acid on kidney GSTs from the saline and corn oil control groups respectively

The IC_50_ values were obtained from Figs. 4–7. Each of the value was obtained from thesigmoidal dose–response curves as the concentration of the inhibitor was increased and are the mean of 4 values

### Effects of *C. zeyheri* extracts on GST activity in vitro

Inhibition of hepatic and kidney GST activity was observed after exposing cytosolic fractions of control samples to increasing concentration of the aqueous or ethanol extracts. *C. zeyheri* ethanol extract was a more potent inhibitor of the hepatic GSTs than the aqueous extract as observed by shown by their IC_50_ values (Fig. 6 and Table 1). Ethacrynic acid was a more potent inhibitor of the liver GSTs than the *C. zeyheri* aqueous extract whilst the ethanol extract caused inhibition to a greater extent than ethacrynic acid in vitro. The ethanol extract also caused greater inhibition of GSTs in the kidneys than the aqueous extract. Ethacrynic acid was, however, a more potent in vitro inhibitor of kidney GSTs compared to both the aqueous and ethanol *C. zeyheri* leaf extracts (Fig. 7 and Table 1).

## Discussion

Glutathione transferases play a significant role in the detoxification and metabolism of many xenobiotic and endogenous compounds and are over-expressed in some cancer cells. These enzymes, therefore, contribute to detoxification of anticancer drugs, leading to drug resistant tumours and, thus, their inhibition has been suggested as an approach to combat GST-induced resistance. Many plant-derived compounds possess anticancer activity and inhibition of GSTs would add to their antitumour potency by preventing drug resistance. Induction of GSTs in vivo is potentially beneficial in protecting the cells from electrophillic insult and electrophillic compounds are deficient in paired electrons thus they can cause diseases such as cancer and neurodegenerative diseases by snatching electrons from macromolecules such as DNA, and proteins [45]. In addition, GSTs also protect cells by sequestering compounds through their ability to bind most of the non-polar and hydrophobic molecules [46]. Inhibition of GST-mediated endogenous reactions may interfere with physiological processes such as the synthesis of prostaglandin D_2_ [41].

*C. zeyheri* aqueous extract caused hepatic (200 mg/kg dose) and renal (50 mg/kg dose and 200 mg/kg dose) increase in GST activity. Natural active compounds derived from plants, such as flavonone and flavones, have been shown to increase GST in vivo activity in rat liver as a result of induction of these enzymes [43]. One of the major mechanisms of protection against carcinogenesis, mutagenesis and other forms of toxicity mediated by carcinogens is the induction of enzymes involved in their metabolism particularly the Phase II enzymes such as the GSTs [5]. Studies have been carried out in which case other plant extracts of different species caused hepatic induction of Phase II enzymes, including the GSTs [44]. The best known among compounds, which preferentially or selectively induce Phase II enzymes are the antioxidants [47, 48]. Antioxidants coordinately elevate GSTs through a common transcriptional activation pathway controlled by an antioxidant response element. This process of induction has been extensively studied in the liver [49]. The mechanism by which *C. zeyheri* caused an increase in GST activity is not known. Induction of GSTs via activation of transcription mechanism may have occurred in the rat liver and kidney tissues since all plants generally contain secondary metabolites and most of these secondary metabolites have been shown to have antioxidant effects [50].

Traditional healers in Zimbabwe, usually administer *C. zeyheri* in aqueous form by suspending the leaves of the plant in water and administering the extract [11]. The leaves are usually targeted as they are the easiest parts of the plant to obtain and they have also been shown in other plants to contain the highest amounts of secondary metabolites. As a result, the in vivo inductive effects of *C.zeyheri* observed in the rat tissues might be expected to occur in other mammalian tissues and if extrapolated to humans, induction of GSTs can be expected to predominate. This could result in beneficiary or adverse effects of *C. zeyheri*. The beneficiary part would be that, the *C. zeyheri* when taken alone would enhance the antioxidant defense mechanisms due to induction of the GSTs which are involved in the detoxification of reactive oxygen species [6]. In the body, GST is involved in the detoxification of hydrogen peroxide by catalysing its reduction to non-toxic products [51]. The effects of free radicals, which play a major role in the pathogenesis of many diseases including atherosclerosis, ischemic heart disease and cancer, can be reduced [52]. Since this plant is used medicinally for rheumatism and joint pain [15], the basis of these uses could be by inhibition of GSTs which through their action could cause a decrease in the synthesis of prostaglandins [41].

The undesirable effects of *C. zeyheri* would be encountered if *C. zeyheri* in aqueous form, is simultaneously taken with a drug that is metabolised by GST. Metabolism of the drug by GST renders the drug more soluble and enhances its excretion from the body [53]. This would in turn reduce the therapeutic effects of the drug or even cause therapeutic failure as the residence time of the drug in the body is reduced. Such herb-drug interactions are likely to occur as this issue has been a cause for concern especially in the rural areas where people use herbal medicines as their primary source of health care and due to poverty. These people cannot easily access conventional medicines regularly and due lack of knowledge about the possible consequences may take different kinds of medicines at the same time [54]. Encouraging these people to take *C. zeyheri* and conventional medicines separately would greatly benefit these people and avoid related misfortunes.

Induction of phase II drug metabolising enzymes is also an important aspect in drug discovery, the ability to induce GST activity is a property common to many chemopreventive agents. The induction of GSTs appears to be an important mechanism for diminishing carcinogenicity. Chemoprevention is one of the most promising areas of cancer research [50]. Potential chemopreventive agents may function by different mechanisms one of which involves the induction of phase II detoxifying enzymes [55]. With regards to this, *C. zeyheri* can be a potential chemopreventive agent as a result of the inductive effects of the aqueous extract on the mammalian GSTs.

No alterations in organ/body weight ratios were observed between the animals administered with the plant extract compared to the control animals. This indicated that there were no significant weight changes of both the organ weight as well as the weight of the animals in general, upon treatment with the plant extract. From these results, it may be concluded that there was no injury that occurred to the organs when the animals were treated with the plant extract. A change in body or internal organ weight is an important index on the adverse side effects of a chemical compound, including a plant extract. A gain in weight by animals during an experimental period may be an indication that the plant extract did not hamper the growth of the animals, whilst a reduced weight of organs, could be as a consequence of leakage of cytosolic enzymes into the extracellular fluid serum. A decrease in the overall body weight of the animal is usually a clear indication of toxicity [56]. With regards to this, *C. zeyheri* did not have any toxic effects on the rats.

The medicinal uses of extracts from *P. curatellifolia* plant are numerous. The plant has been used for wound healing and skin problems, treatment of malaria, typhoid fever, fracture and gastrointestinal disorders, while the leaf and bark extracts are deployed in the treatment of pneumonia, eye and ear diseases, and the roots for treatment of cataracts and ear pain [57] and liver disorders. The seeds of *P. curatellifolia* exert a reduction in the plasma glucose, and the level of low density lipoprotein thus they have anti-diabetic properties. *P. curatellifolia* seeds also have antioxidant properties that play a major role in ameliorating secondary complications resulting from oxidative damage in diabetes [58]. Due to its many uses in traditional medical practices it is of importance to determine the effects of the extracts from this plant on liver and kidney glutathione transferases. The aqueous extract of *P. curatellifolia* had a dose-dependent increasing effect on the liver protein content, especially at the highest dose in the treated rats. However, this could not be related to the decrease in GST activity in the same organ. In a study by Hayeshi et al., [59] most of the compounds from plant extracts they used did not show inhibitory activity towards human GSTs and they suggested that the target of the compounds in vivo were particular resistance proteins in the cell. This might have also have been the case with the *P. curatellifolia* ethanolic leaf extract used in this present study instead of it targeting the GST enzymes. Another explanation for the differences in the performance of GSTs from the livers and kidneys could be because the inhibition of GSTs is substrate-dependent as shown by the inhibition of human recombinant GSTs by antimalarials [60].

The inhibition of GSTs has been extensively studied in vitro and the following compounds from plants have been found to be inhibitors of the enzymes: tannic acid, thonningianin A, cibacron blue, hematin, ethacrynic acid, ellagic acid, ferulic acid, caffeic acid, stilbene, quercetin, chlorogenic acid and curcumin have been long reported by many researchers [61–64]. Ethacrynic acid binds to the H-site of GSTs but has low affinity for the enzyme, and conjugated with GSH, it is easily excreted from the cell. Therefore, the aqueous extracts may have high levels of these as an increase in the dose caused increased enzyme inhibition also.

From the in vitro results obtained in this study, the ethanol extracts was effective in the inhibition of rat liver GSTs than the aqueous extraction. This effect was confirmed by the IC_50_ values. The half maximum inhibitory concentration value (IC_50_) is a measure of the effectiveness of a compound in inhibiting a biological process or biological function by 50 %. The ethanol extract of this plant exhibited GST inhibitory activity in the liver with an IC_50_ value of 12 μg/mL and for the aqueous extract the value was 24 μg/mL. The IC_50_ value of ethacrynic acid, a standard GST inhibitor was found to be 10 μg/mL. This data suggested that the ethanolic extract of *P. curatellifolia* contained some bioactive compounds that inhibited liver GSTs activity in vitro and these were not present in the aqueous extracts. The IC_50_ values of *P. curatellifolia* ethanol extracts on the kidney GSTs and of the aqueous extracts on both the liver and the kidney GSTs were higher than those of ETA, a standard GST inhibitor indicating that this extract was an effective inhibitor. GST Sigma (hematopoietic prostaglandin D_2_ synthase H-PGDS,) is involved in inflammation by its role in the isomerisation of PGH_2_ to produce PGD2, which is an allergic mediator and promotes the inflammatory process [64]. H-PGDS is, thus, a target for the design of anti-inflammatory and anti-allergic drugs. Thus, the inhibition of GSTs by *P. curatellifolia* may validate its use for the treatment of aches and inflammation [21].

*P. curatellifolia* leaf extracts showed significant inhibition of GSTs in vivo. The effects observed in vitro may differ from those effects observed in vivo [65]. Discrepancies between in vitro and in vivo results have been described elsewhere for antioxidants such as ellagic acid and curcumin that are in vitro inhibitors, but in vivo inducers of GSTs [66]. The inhibition might be due to the presence of compounds which decrease the expression of GSTs in vivo which does not occur in vitro [67]. The difference between the in vivo and in vitro effects of plant extracts on GSTs may have been because the inhibition of the enzyme is a time-dependent reaction that needs longer periods to be observed or could be due to formation of metabolites that do not have inhibitory activity. Thus, the decreased GST activity in vivo correlates to the observed inhibition in vitro and this may suggest that the parent phytochemicals in the extracts and not metabolites may be responsible for the inhibitory effects observed in vivo.

Adverse interactions between drugs and herbs have been reported in many studies [29, 68]. The mechanisms of interactions have been reported to be due to the metabolism of different drugs by the same enzyme system. Nakajima et al., [69] proposed that the interaction between the anticoagulant warfarin and indomethacin was because they are metabolized by the same enzyme system CYP2C9 thus, resulting in a metabolic interaction. It has been shown that about 98 % of HIV patients in Zimbabwe use at least one herbal drug together with their antiretroviral therapies [68]. Therefore, the present study demonstrates that there could be significant herb-drug interactions if *P. curatellifolia* leaf extracts are co-administered with other synthetic conventional drugs such as anticancer or anti-HIV drugs. For anti HIV drugs, there would be a significant failure in reducing the viral loads in patients resulting in increased mortality of patients. For anticancer compounds the metabolism of such drugs as cisplatin, busulfan, chlorambucil, cyclophosphamide, melphalan and thiotepa by GSTs would be inhibited by *P. curatellifolia* extracts leading to decreased plasma concentrations of the drugs. In the end this has the effect of increasing the development of drug resistant cancers. This is because drug interactions caused by metabolic processes are regarded as the most important factors that affect the concentrations of drugs in the body [70]. Moreover, the GSTs inhibitory effects of *P. curatellifolia* extracts could be of significant importance in drug-resistant neoplasia as over-expression of GSTs in tumor cell lines is responsible for their resistance to certain chemotherapeutic drugs [71, 72]. Therefore, this means that the use of inhibitors to modulate the activity of GSTs during chemotherapy are a promising strategy in the battle against multi-drug resistance that could result in enhanced therapeutic efficiency of anticancer compounds.

It was also noted that different solvents had an effect on the activities of GSTs. Corn oil which was used as a vehicle for the ethanol extract was shown to increase the activity of GSTs when compared with animals given saline. Saline was shown to alter the activities of the GSTs, depending on whether it was the liver or kidneys. GST activities have been noted to be very high in the liver and less in the extra hepatic organs [73]. Corn oil has been reported to increase the levels of GSTs in rats when compared to animals that were not treated [74]. It is possible the observed effects are due to the different vehicles which were used for the extracts in addition to the effects of the extracts themselves. However, in this study, it is important to note that when comparisons of GST activities were performed, these were determined on animals given the same vehicle for the extracts and, therefore, cancelling out the effects of corn oil.

When drugs are given orally, they are transported to the liver where biotransformation reactions take place. Plant derived phytochemicals when administered in vivo, have been shown to induce xenobiotic metabolism enzymes including glutathione transferases via the antioxidant responsive element [75]. In this study, the effects *C. zeyheri* observed in vivo were dependent on the type of whether the extract was from the aqueous extract (saline as a vehicle) or ethanol extract (corn oil as a vehicle). The aqueous extract group showed increased GST activity regardless of whether the enzyme source was the kidney or liver. In vitro, the opposite was observed and there was decreased GST activity upon incubation with the aqueous extracts. It seems, therefore, that whilst the aqueous extract of *C. zeyheri* had inductive effects in vivo, in vitro there was decreased activity. This suggests that the extracts themselves or some metabolites may have increased the activity of GSTs directly or indirectly (inductive effect). Azizi et al., [76], showed similar results to our study as they found that the *Mitragyna speciose* leaf extract had different effects on GST activity in vivo and in vitro. In their study, they found that there was concentration-dependent inhibition of GST activity in vitro, whilst in vivo there was a general increase in GST activity. Natural dietary compounds have been shown to induce Phase II detoxifying enzymes via gene activation through the Nuclear factor-erythroid 2-related factor (Nrf2) -antioxidant response (ARE) pathway [75].

## Conclusions

In summary, *C. zeyheri* extracts, have an inductive effect on GST activity in vivo when administered in aqueous solution but inhibited GST when administered as an ethanol extracts. *P. curatellifolia* inhibited liver GSTs in vivo when administered as an aqueous solution. Both extracts inhibited GST activity in vitro suggesting that the effects observed increases observed in vivo could be due to the extracts or their metabolites acting on the inductive system of the cells to increase the levels of the enzymes. Induction of GSTs would be cytoprotective against the toxic effects electrophilic chemicals but potentially dangerous outcomes may occur if the activity of GSTs in scavenging electrophilic metabolites is inhibited by these plant species. The inhibition of GSTs by these plant extracts may support their uses in the treatment of fever, pain and inflammation as these enzymes are involved in endogenous activities that produce certain physiological products including pain mediators. There is need to carry out further studies to isolate the bioactive compounds responsible for inhibiting GSTs and identify their structures.
